# Beyond Perceived Evaluation: Interventional Studies of Artificial Intelligence Use in Medical Education

**DOI:** 10.31662/jmaj.2026-0220

**Published:** 2026-08-21

**Authors:** Shigeki Matsubara

**Affiliations:** 1 Department of Obstetrics and Gynecology, Jichi Medical University, Shimotsuke, Japan; 2 Department of Obstetrics and Gynecology, Koga Red Cross Hospital, Koga, Japan; 3 Medical Examination Center, Ibaraki Western Medical Center, Chikusei, Japan

**Keywords:** artificial intelligence, education, evidence, medical education

## To the Editor,

Triggered by the valuable study by Ninomiya et al. ^(1)^, I consider that current evidence on artificial intelligence (AI) use in medical education remains largely perception based; outcome-oriented, quasi-experimental studies are urgently needed.

Ninomiya et al. ^(1)^ showed that many medical students and faculty members reported positive perceived evaluation of AI; however, the respondents expressed concerns about ethical aspects and skill deterioration, and some, especially older staff (considered experienced educators), supported fundamentally prohibiting AI use. Thus, similar to many previous studies, merits and demerits are claimed based on perception. This is inherent to this type of study; Ninomiya et al. ^(1)^ should not be criticized. The following approach may provide a more practical answer.

Evidence on AI use in medicine remains limited ^(2)^, mainly because interventional studies are difficult to conduct. This conceptual approach has been previously proposed in the context of AI use in medical writing ^(3)^ and may be extended to medical education. For example, in the 202X semester, students in a corresponding grade (e.g., second year) and faculty members could be “strongly recommended” to use AI. In 202X+1 (the following year), the subsequent second-year cohort could be “prohibited from using AI as much as possible.” Outcomes selected according to the study aim, such as reported ethical issues, writing ability, students’ evaluation of faculty members, or even students’ examination scores, could then be compared between the cohorts. Despite potential confounders, this approach may provide a pragmatic, policy-level natural experiment.

The key aspect of this study design is that participants cannot be required to “use” or “not use” AI because individuals have the freedom to use AI under institutional regulations. However, we can “recommend” AI use or nonuse; this may not violate research ethics. Even if the study eventually favors AI use, recommending nonuse may still be justifiable because it is currently unknown whether such a recommendation would disadvantage participants. Regardless, careful ethical consideration is needed.

AI may contribute to patients’ future health, where progress is often required rapidly. Unlike such clinical settings, medical education allows time for evaluation. Broadening the view, medical education might be a good platform for considering AI use in medicine.

Ninomiya et al. ^(1)^ concluded that future studies with larger and more diverse samples will be necessary; I totally agree. Beyond “perceived” evaluation, we should step forward to “objective” evaluation of outcomes. Ethical considerations for such research and detailed study design require multidisciplinary knowledge. I hope for a study directly addressing the outcomes of AI use.

## Article Information

### Author Contributions

Shigeki Matsubara designed this study, and wrote and edited the manuscript.

### Conflicts of Interest

None

### Ethics Approval

Jichi Medical University does not require institutional review board approval for this type of article. Patient anonymity preservation and informed consent for publication are not applicable.

## References

[1] Ninomiya S, Yamamoto K, Mieno E, et al. Current status of generative artificial intelligence utilization in medical education: a cross-sectional survey of medical students and faculty. JMA J. 2026;9(4):742-9. 10.31662/jmaj.2025-0580 PMC13453582 42577023

[2] Matsubara S. Artificial intelligence in medical writing: is it an exception to evidence-based medicine? JMA J. 2026;9(1):369-71. 10.31662/jmaj.2025-0443 PMC12889114 41676812

[3] Matsubara S. A hypothetical experiment: evaluating the real-world impact of artificial intelligence use in academic writing. JMA J. 2025;8(2):662-3. 10.31662/jmaj.2024-0411 PMC12095812 40415988

